# A Case–Control Study of the Effects of Chronic Low Back Pain in Spatiotemporal Gait Parameters

**DOI:** 10.3390/s21155247

**Published:** 2021-08-03

**Authors:** Aurora Castro-Méndez, Inmaculada Requelo-Rodríguez, Manuel Pabón-Carrasco, María Luisa González-Elena, José Antonio Ponce-Blandón, Inmaculada Concepción Palomo-Toucedo

**Affiliations:** 1 Podiatry Department, University of Seville, 41009 Seville, Spain; maruchi1@us.es (M.L.G.-E.); ipalomo@us.es (I.C.P.-T.); 2 Clinica Pie y Deporte, 41018 Seville, Spain; inmarequelo@hotmail.com; 3 Spanish Red Cross Nursing School, University of Seville, 41009 Seville, Spain; mpabon2@us.es (M.P.-C.); japonce@cruzroja.es (J.A.P.-B.)

**Keywords:** foot, low back pain, physical activity, OptoGait

## Abstract

Chronic low back pain and biomechanical walking imbalances are closely related. It is relevant to identify if there are alterations in spatiotemporal gait patterns in subjects with CLBP (cases) versus healthy subjects (controls) to plan training interventions of motor control gait patterns, and thus allowing normal physical activity of the individual. This study is intended to identify if spatiotemporal alterations occur in the gait cycle in CLBP subjects (cases) compared with a control group (healthy patients) analyzed with an OptoGait LED sensors gait program. Method: A total of *n* = 147 participants: *n* = 75 cases (CLBP) and *n* = 72 healthy controls subjects were studied with OptoGait gait program. Results: Significant differences were found between the two groups and both feet in foot stride, for the differences of the total stride and contact, for gait cadence and total stride length of the gait cycle (*p* < 0.05). Conclusions: CLBP may alter some normal gait patterns measured by OptoGait; this finding presents imbalances in gait cycle as an underlying factor. The gait is part of daily life of any individual and it is an important physical activity in relation to the maintenance of an optimal state of health. In addition, future studies are deemed necessary.

## 1. Introduction

Mechanical low back pain is a very common, expensive, and significant health issue in the western world. The role of foot posture and leg length discrepancy in contributing to abnormal biomechanics of the lumbopelvic region and low back pain is not sufficiently investigated. Chronic low back pain is characterized by its development in a period of more than 3 months and being the second cause of work disability worldwide. Its approach is complex and sometimes its etiological factor cannot be identified, attributing it to chronic nonspecific low back pain (CLBP) [1,2].

Various research argues that subjects with CLBP modify spatiotemporal gait parameters (step length, stride length, speed or cadence), altering this balance as a compensation mechanism for antalgic purposes [2,3,4,5], consequently, making the subject fearful and gait unstable, possibly creating a predisposition to the negative effects of a sedentary life (obesity, diabetes, depression, etc.) [2,3,4,5,6,7].

Balance and deficit in walking are some of the main characteristics of aging and are considered among the risk factors for falls [8]. Postural control is maintained by continuous equilibrated contraction of anti-gravitational body musculature. Alterations in the position of several parts of the body may lead to variations of load due to the acceleration of gravity and influence the plantar pressure [9].

The posture of the foot can cause orthostatic imbalances or may cause pathologic modifications in other parts of the body (oculomotor system, stomatognathic system, etc.). Bricot [10] states that postural modifications may have either an upward progression with a plantar starting point or a downward path beginning from the head (eyes, temporomandibular joints, vestibular system). Therefore, there are many studies that use muscle chains to assess the impact of foot posture on other systems or joints and vice versa [10,11].

On the other hand, the current trend in the approach of CLBP is rehabilitation by performing physical activity, thanks to its benefits (psychological, cardio-vascular, weight management, muscle stabilization) [7,12]. Living an active life seems to prevent the onset of CLBP and the negative effects of a sedentary lifestyle (obesity, depression, diabetes, osteoarticular problems, etc.) [12].

It is considered relevant to study in depth the spatiotemporal gait imbalances produced in CLBP to implement interventions that allow to restore balance and physical activity, increasing the health level of the individual [7,12,13,14,15]. The aim of this study is to identify whether alterations of spatiotemporal gait patterns occur in subjects with CLBP versus healthy subjects using the OptoGait system. The hypothesis of this research is that pain occurring in CLBP predisposes to a modification of gait cycle parameters.

## 2. Materials and Methods

### 2.1. Trial Design

A case–control, cross-sectional study: a group of subjects with CLBP (cases) was compared in relation to the spatiotemporal gait parameters evaluated with an OptoGait optical sensor system with a treadmill program lasting 30 sec at a comfortable walking speed for the subject compared with healthy subjects (controls).

### 2.2. Participants

A total of *n* = 147 participants: *n* = 75 cases and *n* = 72 healthy controls recruited from the Clinical Area of Podiatry (ACP) from the University of Seville from November 2020 to March 2021.

### 2.3. Inclusion Criteria

All the participants were healthy adult patients going to the ACP during the recruitment period of this study, consulting for common podiatric conditions, such as skin and nail disorders. The patients who had been diagnosed of nonspecific CLBP by a specialist or family physician composed the case group.

### 2.4. Exclusion Criteria

A serious illness, pregnancy, back or/and osteoarticular lower limb surgery, musculoskeletal disorders, severe morphological deformity of the foot, current treatment with orthoses, or non-idiopathic CLBP. 

### 2.5. Procedures

**Figure 1:**
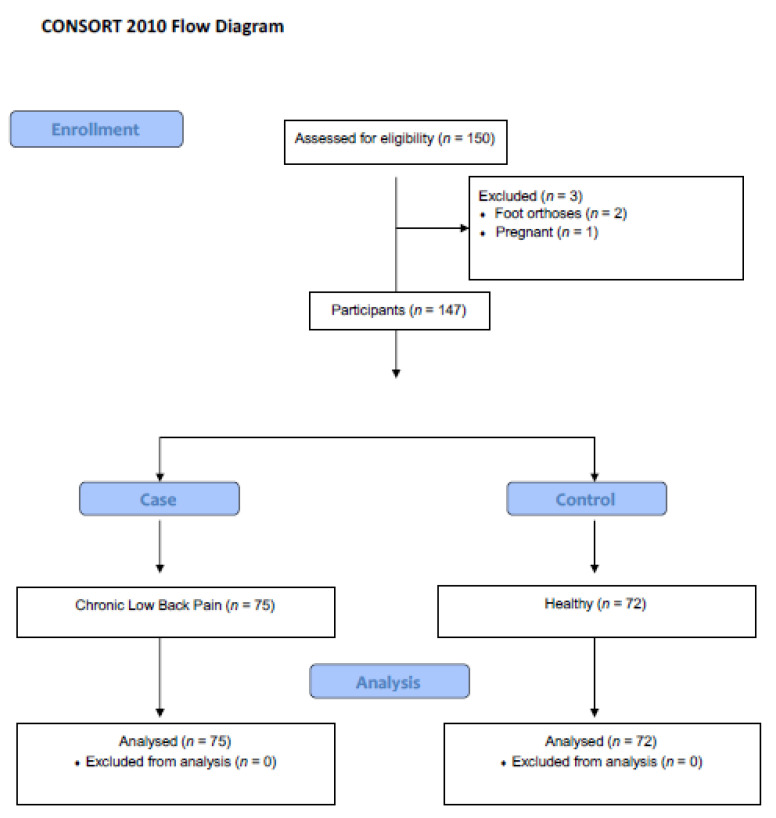
CONSORT flow diagram.

Once the subject agreed to voluntarily participate in the research, an interview was conducted where the appertaining data was collected by a research assistant (IRR) (Figure 1, CONSORT Strengthening the Reporting of Observational Studies). The demographic data collected were: sex, age, mass and height. Body mass index (BMI) was calculated. The CLBP was measured only for the case group using a visual analogue scale (VAS) and with the Oswestry Disability Questionnaire (ODI) for lower back pain on the day they were recruited for the research. Previously, patients had a medical diagnosis of chronic pain (pain for more than six months) [16,17].

### 2.6. Ethical Statements

All subjects gave their informed consent for inclusion in the research, and the study was conducted in accordance with the Declaration of Helsinki, and the protocol was approved by the Ethics Committee of Seville (0966-N-20). The research followed the guidelines of the Declaration of Helsinki [18], and it was registered according to the guideline of the Declaration STROBE [19].

The subjects’ gaits were analyzed on a treadmill where the OptoGait Photocell Sensor System (Version 1.6.4.0, Microgate, Bolzano, Italy) was installed. The treadmill gait test program was used at a comfortable walking speed for the participant, for which the subject was previously asked to walk at the speed that was most comfortable and safe for to them). The subjects were asked to walk beforehand and they indicated when they were comfortable on the same day of the test. However, there are studies that indicate comfort patterns with respect to speed.

Sensors technology is used to evaluate the kinematics of gait and physical activity (mobile phones, accelerometers, video recording systems, three-dimensional sensors, etc.) [5,20,21]. The OptoGait sensor system is commonly used to accurately monitor spatiotemporal gait patterns and, thus, compensating for alterations involving a deficit in motor control and balance before and after an intervention [2,5,7,22,23].

Once it was detected, it was the one that was considered during the 30 s (sec) that lasted the measurement procedure according to protocol [11]. The OptoGait optical data sensor system was used for its reliability [2,24]. Although the subjects walked for two minutes until they adapted to the situation, only the last 30 s were evaluated. Minimizing the measurement time was avoided to prevent falls and pain.

**Figure 2:**
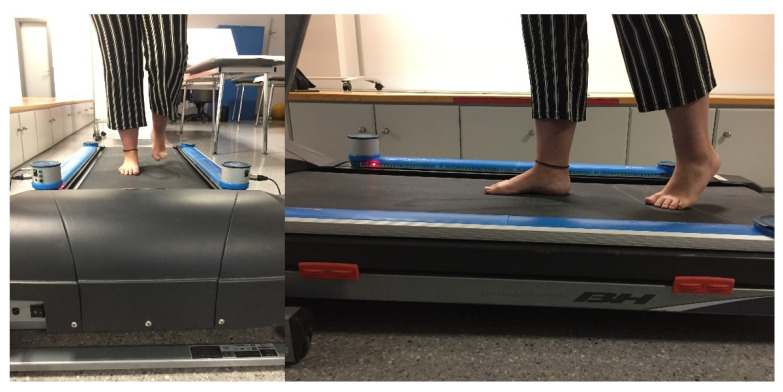
Gait analysis with OptoGait.

The two photocell systems were placed on the treadmill and in all cases the subject walked barefoot (Figure 2). The analysis using this system is relevant as it allows to test kinematic anomalies detected in these parameters: right and left foot step length in centimeters (cm), step duration—the time elapsed from the initial ground contact of the first foot to the initial ground contact of the opposite foot—in seconds and percentage (%) for each foot, walking cycle duration in seconds (median), cadence or steps per minute (s.p.m.) and total stride length in centimeters (cm). As was described in other studies, two measurements were carried out: an initial one where the subject became familiar with the system and indicated the proper walking speed for them, and a second measurement that was considered in this investigation [2,5].

The sample size was calculated for a power of 0.90 and an alpha error of 0.05 and a size effect of 0.5 (test family: test T, G* Power 3.0.10, Franz Faul, University of Kiel, Kiel, Germany) [25]. A total sample containing 70 subjects was considered necessary for each group. A total of 150 subjects were initially recruited. In the end, 3 subjects did not meet the criteria for inclusion in the investigation.

### 2.7. Statistical Analysis 

The Kolmorov–Smirnov test was used to assess the normality of the variables. For quantitative variables, the average and standard deviations were expressed for each group: age, gender, BMI baseline. Student’s t-test was applied in respect to the different averages. Secondary outcomes were tested for differences by Pearson’s Chi^2^ or the Student’s *t*-test. 

For the statistical analysis, the IBM SPSS Statistics^®^ 24.0 program was performed. A *p* value < 0.05 was considered as statistically significant.

## 3. Results

### 3.1. Description of the Total Sample and by Groups

A total of 147 participants were included (67 men and 66 women). The average age was 49.56 ± 6.31 years (range 20–49 years old). The CLBP for the case group was of 6.59 ± 0.20 median ± DS for VAS and of 33.71 ± 0.20 for ODI.

An analysis was conducted for the variables gender and BMI for the total sample, both for the case and the control groups; the data is shown below (Table 1).

Table 1 shows the sample is not homogeneous with respect to gender and BMI. The next analysis shows the descriptive results for the gait parameters between both groups (Table 2). 

The number of women in the control group was higher than that of men, and compared with the BMI, it appeared to show higher values in the case group (CLBP).

### 3.2. Statistical Analysis

The results have shown a statistical significance for the variables: right and left foot stride time expressed in total %, for the difference of the total stride and contact, for gait cadence and total stride length and of the gait cycle (in all cases *p* < 0.05). 

The step length of the right and left foot in cm for the case group was greater; on the other hand, the step time duration in seconds for both feet and the gait cycle were shorter than the control group.

No differences were found between the laterality of the limbs of each group.

## 4. Discussion

The objective of this study was to identify if there are alterations in spatiotemporal gait patterns in subjects with CLBP compared with a group of healthy subjects assessed using the OptoGait optical sensor system.

Based on the results obtained, statistically significant differences were observed when comparing step time duration (%) in the subject group with CLBP with the group of healthy subjects. A decrease in duration in % was observed in the group of subjects with CLBP; the time elapsed from the initial ground contact of the first foot to the initial ground contact of the opposite foot was reduced significantly compared with that of the group of healthy subjects, and step length was increased. Subjects with CLBP walked more slowly and the cadence decreased significantly compared with healthy controls (in all cases *p* < 0.05).

In general, the CLBP group showed a longer stride and step length for both feet, and a shorter duration of the gait cycle and cadence; in contrast, for the group of healthy subjects the contact times with the ground were longer. Therefore, people with CLBP tend to avoid pain by decreasing the time of limb support. They take long strides but the contact time of each foot is short and the number of steps per second or cadence is low. Nevertheless, biomechanical adaptations may depend on the intensity of symptom.

The data obtained is consistent with other research where it was reported that gait parameters were altered in subjects with CLBP for fear of pain or by an unstable gait [2,5,17,21,26,27].

A study was conducted by Hollman et al. in which they recruited a sample of adults from 70 to 89 years old (108 men and 189 women) and had the subjects walked on a GAITRite^®^ walkway placed on the ground. After the analysis, they concluded that the subjects with CLBP showed different spatiotemporal gait characteristics compared with the group of healthy control subjects. These parameters were significant regarding lower average speed, higher cadence, step duration, and stride length [26]. The age of the sample was higher than that of this research and the gait was carried out on the ground, not on a treadmill.

Another study of Demirel et al. analyzed the gait with OptoGait on a treadmill and compared two groups of adults from 25 to 65 years [2]. They assessed whether the spatiotemporal parameters of the gait were modified between groups: cases (subjects with CLBP who attended the physiotherapy unit) and controls (healthy subjects who were caregivers). They concluded that the modifications were influenced by the degree of disability (assessed with ODI). They argued that this syndrome may result in minor changes in gait patterns in subjects with greater disabilities (ODI value 60%–80%) compared with a more moderate one (ODI value 20%–40%). They observed a temporary reduction in the support phase and an increase in the oscillation phase in CLBP, a higher cadence and lower gait speed; their conclusions were in line with our results except for the cadence in spm. It is important to emphasize that the sample of the Demirel et al.’s study were patients attended in a rehabilitation service specialized in the therapeutic approach of CLBP and presented higher values on ODI than those shown here [2].

Barzylai et al. found some results contradictory to ours. They claimed that subjects with CLBP walked at a lower speed, had a greater asymmetry of movements between two feet and that their step length was shorter. They assessed the kinematic parameters before and after analyzing the effect of a biomechanical device based on a functional rehabilitation treatment program at home and observed that gait patterns had improved, defined by a higher gait speed and a longer step length. A small cohort was used. The analysis was performed with the GaitMat™ system (sensor system set up on the ground on which subjects walk on) [15]. They claimed that after 6 months of treatment, patients normalized their gait patterns; improvements in the gait pattern were accompanied by reduced pain and improved function and quality of life.

Based on the results obtained, it is reasoned that CLBP produces alterations in spatiotemporal gait parameters analyzed with OptoGait, resulting in a reduction of contact of both feet with the ground in %, an increase in step length, and a slower gait speed in contrast to healthy subjects. This suggests that the case group takes steps to avoid pain by increasing the length of the step and the duration of the swing phase to reduce foot contact with the ground. This fact prompts an asymmetry between the right and left feet of the same subject as described in other studies [14,15]. Sometimes, walking on a treadmill can show results that are different from those obtained through walking on another type of surface, as some studies defend [14]. Adaptive measures are therefore taken in motor control as a protective strategy to avoid pain [27].

With regard to the significant difference in BMI between groups, a value of 25.25 (3.61) was assessed for the case group (values greater than 25 mg/kg^2^ indicates overweight) [2,28]. Obesity is associated with an increased risk of musculoskeletal diseases such as low back pain [28]. It is a fact that in the case group, the BMI levels presented overweight values which can influence or be a consequence of CLBP because pain limits the development of physical activity.

As discussed above, the current trend in approaching CLBP is rehabilitation through exercise that can improve CLBP pain. This fact is considered relevant because gait is part of daily life of the individual, and it is an important physical activity in relation to the maintenance of an optimal state of health [28,29,30].

As a result, assessing gait-altering factors can help improve CLBP. Sensor-based technology is a practical way to detect the decline in gait and balance [28,29,30]. The assessment and treatment of CLBP is important; the analysis of factors that can improve this syndrome is considered essential to prevent gait adjustments and to promote earlier intervention and reduce sedentary lifestyles. These interventions may include rehabilitation with physical exercise, the implementation of diets to achieve a healthy weight, and controlling foot imbalances that may affect CLBP [12,15,31,32,33,34].

## 5. Conclusions

The spatiotemporal parameters of the gait cycle evaluated by an optical system of sensors using OptoGait undergo significant modifications in a group of pronator subjects (cases) compared with a group with a normal foot posture index (controls) in relation to the stride length for the right and left foot, for the stride time of each foot, for the duration of the gait cycle and the cadence.

### 5.1. Study Limitations

There have been several limitations in this study: on one hand, the heterogeneity with respect to gender, and on the other, the recruitment of this research is quite different with respect to others described in the literature. In this particular case, the reason for consultation of the recruited subjects was not CLBP. Regarding gender, women are more likely to suffer from foot problems and they demand podiatric treatment more frequently. In this study, significant difference in gender between groups can be a selection bias to consider.

The recruitment of this sample was of subjects attending the Clinical Area of Podiatry of the University of Seville for problems related to the foot. This can be considered decisive in that CLBP did not present very high values for VAS and ODI and can therefore justify not being representative of the population affected by CLBP. Hence, future studies are necessary.

### 5.2. Practical Implications of the Study

Assessing gait disorders may prevent sedentary lifestyle by applying routines that improve chronic lower back pain, and detecting the altered gait parameters may focus on the rehabilitation strategies.

## Figures and Tables

**Table 1:** Whole sample demographic values of the demographic variables for both groups (average ± standard deviation) are shown.

Sample *n* = 147		Group		
		Case *n* = 75	Control *n* = 72	*p*-Value
Gender Male	75 (51.0%)	45 (60.0%)	30 (40.0%)	*p* = 0.007 ^a^
Gender Female	72 (49.0%)	29 (40.27%)	43 (59.72%)	*p* = 0.007 ^a^
BMI	24.00 (2.80)	25.25 (3.61)	22.02 (4.15)	*p* = 0001 ^b^

^a^ Chi-squared test. ^b^ Mann–Whitney U-test. Values are presented as median (interquartile ranges). BMI: body mass index.

**Table 2:** Descriptive statistical analysis after the OptoGait sensor gait analysis between the control and case groups.

Sample *n* = 147		Group		
		Case *n* = 75	Control *n* = 72	*p*-Value
Right foot stride length, cm	60.50 (3.00)	60.70 (2.80)	60.40 (4.15)	*p* = 0.901
Left foot stride length, cm	60.10 (4.40)	60.30 (6.50)	59.90 (2.70)	*p* = 0.438
Ground contact time:				
Right foot sec	0.54 (0.03)	0.54 (0.03)	0.54 (0.09)	*p* = 0.332
Step %	70.50 (6.70)	65.00 (9.30)	71.10 (1.40)	*p = 0.001*
Left foot sec	0.54 (0.03)	0.54 (0.06)	0.55 (0.06)	*p* = 0.101
Step %	70.10 (7.60)	66.00 (10.47)	70.80 (1.00)	*p = 0.001*
Difference stride%	0.10 (3.30)	−0.70 (3.40)	−0.93 (3.00)	*p = 0.001*
Difference contact%	−0.70 (1.50)	−0.70 (2.10)	−0.60 (1.10)	*p* = 0.050
Gait cycle sec	1.10 (0.05)	1.09 (0.06)	1.11 (0.06)	*p* = 0.867
Gait cadence spm	107.90 (9.40)	107.03 (8.70)	110.90 (8.80)	*p* = 0.013
Total Stride length cm Speed, Km/h	119.90 (6.20) 4.33 (0.74)	122.50 (8.45) 4.21 (0.82)	119.50 (3.30) 4.38 (0.92)	*p* = 0.036 *p* = 0.043

Values are presented as median (interquartile ranges). The Mann–Whitney U-Test was used. Significance set at *p* < 0.05.

## Data Availability

Not applicable.

## References

[1] Hartvigsen J., Hancock M., Kongsted A., Louw Q., Ferreira M.L., Genevay S., Hoy D., Karppinen J., Pransky G., Sieper J. (2018). What low back pain is and why we need to pay attention. Lancet.

[2] Demirel A., Onan D., Oz M., Ozel Aslıyuce Y., Ulger O. (2020). Moderate disability has negative effect on spatiotem-poral parameters in patients with chronic low back pain. Gait Posture.

[3] Shin S., Lee M., Song C., Lee K., Shin D. (2014). Agreement between the spatio-temporal gait parameters from treadmill-based photoelectric cell and the instrumented treadmill system in healthy young adults and stroke patients. Med. Sci. Monit..

[4] Hirase T., Okubo Y., Sturnieks D.L., Lord S.R. (2020). Pain Is Associated with Poor Balance in Community-Dwelling Older Adults: A Systematic Review and Meta-analysis. J. Am. Med. Dir. Assoc..

[5] Requelo-Rodríguez I., Castro-Méndez A., Jiménez-Cebrián A., González-Elena M., Palomo-Toucedo I., Pabón-Carrasco M. (2021). Assessment of Selected Spatio-Temporal Gait Parameters on Subjects with Pronated Foot Posture on the Basis of Measurements Using OptoGait. A Case-Control Study. Sensors.

[6] Lansing J.E., Ellingson L.D., DeShaw K.J., Cruz-Maldonado G., Hurt T.R., Meyer J.D. (2021). A qualitative analysis of barriers and facilitators to reducing sedentary time in adults with chronic low back pain. BMC Public Health.

[7] Schega L., Kaps B., Broscheid K.-C., Bielitzki R., Behrens M., Meiler K., Drange S., Franke J. (2021). Effects of a multimodal exercise intervention on physical and cognitive functions in patients with chronic low back pain (MultiMove): Study protocol for a randomized controlled trial. BMC Geriatr..

[8] Maranesi E., Riccardi G.R., Lattanzio F., Di Rosa M., Luzi R., Casoni E., Rinaldi N., Baldoni R., Di Donna V., Bevilacqua R. (2020). Randomised controlled trial assessing the effect of a technology-assisted gait and balance training on mobility in older people after hip fracture: Study protocol. BMJ Open.

[9] Martínez-Jiménez E.M., Losa-Iglesias M.E., Díaz-Velázquez J.I., Becerro-De-Bengoa-Vallejo R., Palomo-López P., Calvo-Lobo C., López-López D., Rodríguez-Sanz D. (2019). Acute Effects of Intermittent Versus Continuous Bilateral Ankle Plantar Flexor Static Stretching on Postural Sway and Plantar Pressures: A Randomized Clinical Trial. J. Clin. Med..

[10] Bricot B. (2020). La Reprogrammation Posturale Globale (Global Postural Reprogramming).

[11] Iacob S., Chisnoiu A., Buduru S., Berar A., Fluerasu M., Iacob I., Objelean A., Studnicska W., Viman L. (2021). Plantar Pressure Variations Induced by Experimental Malocclusion—A Pilot Case Series Study. Healthcare.

[12] Hartvigsen J., Morsø L., Bendix T., Manniche C. (2010). Supervised and non-supervised Nordic walking in the treatment of chronic low back pain: A single blind randomized clinical trial. BMC Musculoskelet. Disord..

[13] Rum L., Brasiliano P., Vannozzi G., Laudani L., Macaluso A. (2021). Non-specific chronic low back pain elicits kinematic and neuromuscular changes in walking and gait termination. Gait Posture.

[14] Koch C., Hänsel F. (2018). Chronic Non-specific Low Back Pain and Motor Control during Gait. Front. Psychol..

[15] Barzilay Y., Segal G., Lotan R., Regev G., Beer Y., Lonner B.S., Mor A., Elbaz A. (2015). Patients with chronic non-specific low back pain who reported reduction in pain and improvement in function also demonstrated an improvement in gait pattern. Eur. Spine J..

[16] Bijur P., Silver W., Gallagher E. (2001). Reliability of the visual analog scale for measurement of acute pain. Acad. Emerg. Med..

[17] Chapman J.R., Norvell D.C., Hermsmeyer J.T., Bransford R.J., Devine J., McGirt M.J., Lee M.J. (2011). Evaluating Common Outcomes for Measuring Treatment Success for Chronic Low Back Pain. Spine.

[18] Declaración de Helsinki de la AMM. Principios Éticos para las Investigaciones Médicas en seres Humanos. Asamblea General de la AMM, Fortaleza, Brasil, Octubre de 2013. https://www.wma.net/es/policies-post/declaracion-de-helsinki-de-la-amm-principios-eticos-para-las-investigaciones-medicas-en-seres-humanos/.

[19] Vandenbroucke J.P., Von Elm E., Altman D.G., Gotzsche P.C., Mulrow C.D., Pocock S., Poole C., Schlesselman J.J., Egger M., Strobe Initiative (2007). Strengthening the Reporting of Observational studies in Epidemiology (STROBE): Explanation and elaboration. Epidemiology.

[20] Migueles J., Cadenas-Sanchez C., Alcantara J., Leal-Martín J., Mañas A., Ara I., Glynn N., Shiroma E. (2021). Calibration and Cross-Validation of Accelerometer Cut-Points to Classify Sedentary Time and Physical Activity from Hip and Non-Dominant and Dominant Wrists in Older Adults. Sensors.

[21] Chan H., Zheng H., Wang H., Newell D. Assessment of gait patterns of chronic low back pain patients: A smart mobile phone based approach. Proceedings of the 2015 IEEE International Conference on Bioinformatics and Biomedicine (BIBM).

[22] Jaén-Carrillo D., García-Pinillos F., Cartón-Llorente A., Almenar-Arasanz A.J., Bustillo-Pelayo J.A., Roche-Seruendo L. (2020). Test–retest reliability of the OptoGait system for the analysis of spatiotemporal running gait parameters and lower body stiffness in healthy adults. Proc. Inst. Mech. Eng. Part P J. Sports Eng. Technol..

[23] User Manual. Microgate, Bolzano, Italia OptoGait. http://www.optogait.com/OptoGaitPortal/Media/Manuals/Manual-ES.PDF.

[24] Simoni L., Scarton A., Macchi C., Gori F., Pasquini G., Pogliaghi S. (2021). Quantitative and Qualitative Running Gait Analysis through an Innovative Video-Based Approach. Sensors.

[25] Faul F., Erdfelder E., Buchner A., Lang A.-G. (2009). Statistical power analyses using G*Power 3.1: Tests for correlation and regression analyses. Behav. Res. Methods.

[26] Hollman J.H., McDade E.M., Petersen R.C. (2011). Normative spatiotemporal gait parameters in older adults. Gait Posture.

[27] Henchoz Y., Soldini N., Peyrot N., Malatesta D. (2015). Energetics and mechanics of walking in patients with chronic low back pain and healthy matched controls. Graefe’s Arch. Clin. Exp. Ophthalmol..

[28] Koremans F., Chen X., Das A., Diwan A. (2021). Changes in Back Pain Scores after Bariatric Surgery in Obese Patients: A Systematic Review and Meta-Analysis. J. Clin. Med..

[29] Maki B.E. (1997). Gait Changes in Older Adults: Predictors of Falls or Indicators of Fear?. J. Am. Geriatr. Soc..

[30] Muchna A., Najafi B., Wendel C.S., Schwenk M., Armstrong D.G., Mohler J. (2018). Foot Problems in Older Adults. J. Am. Podiatr. Med. Assoc..

[31] Castro-Méndez A., Munuera P.V., Albornoz-Cabello M. (2013). The short-term effect of custom-made foot orthoses in subjects with excessive foot pronation and lower back pain. Prosthetics Orthot. Int..

[32] Menz H., Alyssa B., Riskowski J., Howard J., Hannan T. (2013). Foot posture, foot function and low back pain: The Framingham foot study. Rheumatology.

[33] Yazdani S., Dizji E., Alizadeh F., Hassanlouei H. (2018). Effect of chronic idiopathic low back pain on the kinetic gait characteristics in different foot masks. J. Biomech..

[34] Cambron J.A., Duarte M., Dexheimer J., Solecki T. (2011). Shoe Orthotics for the Treatment of Chronic Low Back Pain: A Randomized Controlled Pilot Study. J. Manip. Physiol. Ther..

